# Removal of biogenic compounds from the post-fermentation effluent in a culture of *Chlorella vulgaris*

**DOI:** 10.1007/s11356-019-05162-6

**Published:** 2019-04-29

**Authors:** Karolina Szwarc, Dawid Szwarc, Marcin Zieliński

**Affiliations:** Department of Environmental Sciences, Department of Environmental Engineering, University of Warmia and Mazury in Olsztyn, Ul. Warszawska 117A, 10-720 Olsztyn, Poland

**Keywords:** *Chlorella vulgaris*, Post-fermentation effluent, Microalgae, Biomass

## Abstract

Wastewater rich in organic carbon, nitrogen and phosphorus may serve as a convenient source of carbon and nutrients for a year-long microalgae production. Scientific reports indicate that some single-cell microalgae such as *Chlorella* and *Scenedesmus*, are highly tolerant to wastewater environments and efficiently remove biogenic compounds. The aim of this study was to determine the possibility of using the effluent produced in the process of anaerobic degradation of whey as a culture medium for the multiplication of *Chlorella vulgaris* algae biomass and to characterise their growth efficiency and rate. The content of nitrogen and phosphorus in wastewater was sufficient for conducting an effective culture of algae. The efficiency of nitrogen removal in the flow system was 15.61 ± 1.38 mg N/dm^3^/day.

## Introduction

In recent years, anaerobic wastewater treatment originating from a variety of industries has been regarded as an economically and technologically justified process (Tiwary et al. 2015). The anaerobic microbiological process, where complex organic substances are transformed into methane and carbon dioxide, is a widely applied technology for the stabilisation of waste with a concurrent generation of energy in the form of biogas (Niu et al. 2014). Although the process of methane fermentation offers high efficiency in the removal of organic compounds, nutrients such as nitrogen and phosphorus are removed to a small degree.

Despite many advantages, anaerobic technologies of wastewater treatment also feature certain imperfections, which limit the possibilities of common application (Chan et al. 2009). One of the drawbacks of the fermentation process is the removal of biogenic compounds (nitrogen and phosphorus) solely through sludge biomass growth. These compounds are removed in low amounts which usually do not exceed 10% (Jędrzejewska-Cicińska and Krzemieniewski 2010). Therefore, anaerobic reactors are systems which do not ensure a comprehensive removal of contamination. Wastewater treated in this way does not meet the criteria for being discharged directly to a recipient. Therefore, it requires additional technological treatment which generates further exploitation costs. For this reason, it is necessary to search for solution to improve the efficiency of anaerobic technologies and make them more universal. One such solution could be phytoremediation, which uses plants to neutralise contaminations (Oswald 2003). Wastewater rich in organic carbon, nitrogen and phosphorus may serve as a convenient source of carbon and nutrients for a year-long microalgae production (Schenk et al. 2008). Scientific reports indicate that some single-cell microalgae such as *Chlorella* and *Scenedesmus* are highly tolerant to wastewater environments and efficiently remove biogenic compounds (Ruiz-Marin et al. 2010).

Microalgae constitute a diversified group of eukaryotic photosynthetic microorganisms which colonise both the marine and freshwater environments. Microalgae are among the fastest developing photosynthetic organisms. Their photosynthetic mechanism is similar to that of terrestrial plants. Microalgae do not compete for cultivated land. With access to water, carbon dioxide and biogenic compounds such as nitrogen and phosphorus, offer higher biomass yields than terrestrial plants. Algae have the ability to produce 50 times more biomass than higher plants (Li et al. 2008; Apt and Behrens 1999). Different varieties of algae are able to develop in a wide variety of environments, even in degraded or contaminated areas (Mata et al. 2010). Such cultures bring a positive effect to the natural environment because algae may be produced using communal, agricultural or industrial waste-water containing carbon dioxide which is required for their growth CO_2_ (Chisti 2007). The molar ratio of the algae biomass main components proposed by Grobbelaar (2003) is as follows: CO_0.48_H_1.83_N_0.11_P_0.01_. In the course of microalgae population development, four following phases can be distinguished: adaptation phase, growth phase, stationary phase and decline phase (Barsanti and Gualtieri 2006; Singh et al. 2014).

The importance and interest in algae has been increasing with time. More and more frequently algae cultivations of high purity are carried out (Lorenz and Cysewski 2000). Substances obtained from algae may constitute a source of nutritional value and a diet component for both humans and animals (Dallaire et al. 2007). Microalgae have already been used as diet supplements, an addition to cosmetics, for wastewater treatment and as a potential biomass source in the production of biofuels (Aslan and Kapdan 2006; Feng et al. 2011; Gellenbeck 2012). The benefits of using wastewater as a culture medium for microalgae production include a reduction in water use and costs of nutritional components added to cultures and the removal of nitrogen and phosphorus from wastewater (Pittman et al. 2011). There are few reports regarding the use of anaerobic fermentation effluents for cultivation of *Chlorella vulgaris*. These studies have focused mainly on the production of algae biomass in the static conditions. In such an arrangement, the researchers added a single dose of the anaerobic effluent and performed an algae cultivation.

There are no reports on algae cultivation with the use of anaerobic effluents in dynamic conditions. Therefore, the author of this publication performed an experiment on algae cultivation in dynamic conditions with simultaneous inflow of anaerobic effluent and biomass collection.

The aim of this study was to determine the possibility of using the effluent produced in the process of anaerobic degradation of whey, as a culture medium for the multiplication of *C. vulgaris* algae biomass and to characterise their growth efficiency and rate.

## Materials and methods

### Microorganism and culture medium

**Fig. 1:**
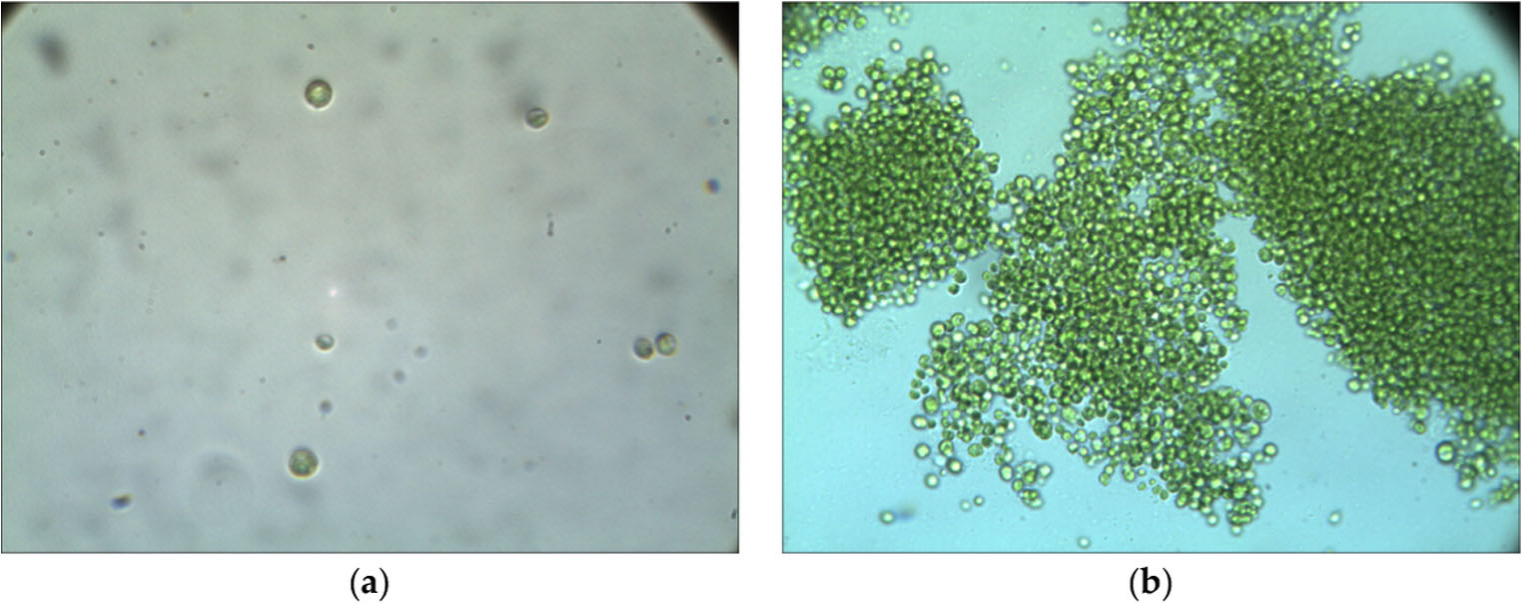
*Chlorella vulgaris* seen under microscopic magnification: **a** ×100; **b** ×40

The study used *C. vulgaris* microalgae originating from a culture of the *Collection of Baltic Algae* of the Institute of Oceanography at the University of Gdańsk (Fig. 1).

### Research station

**Fig. 2:**
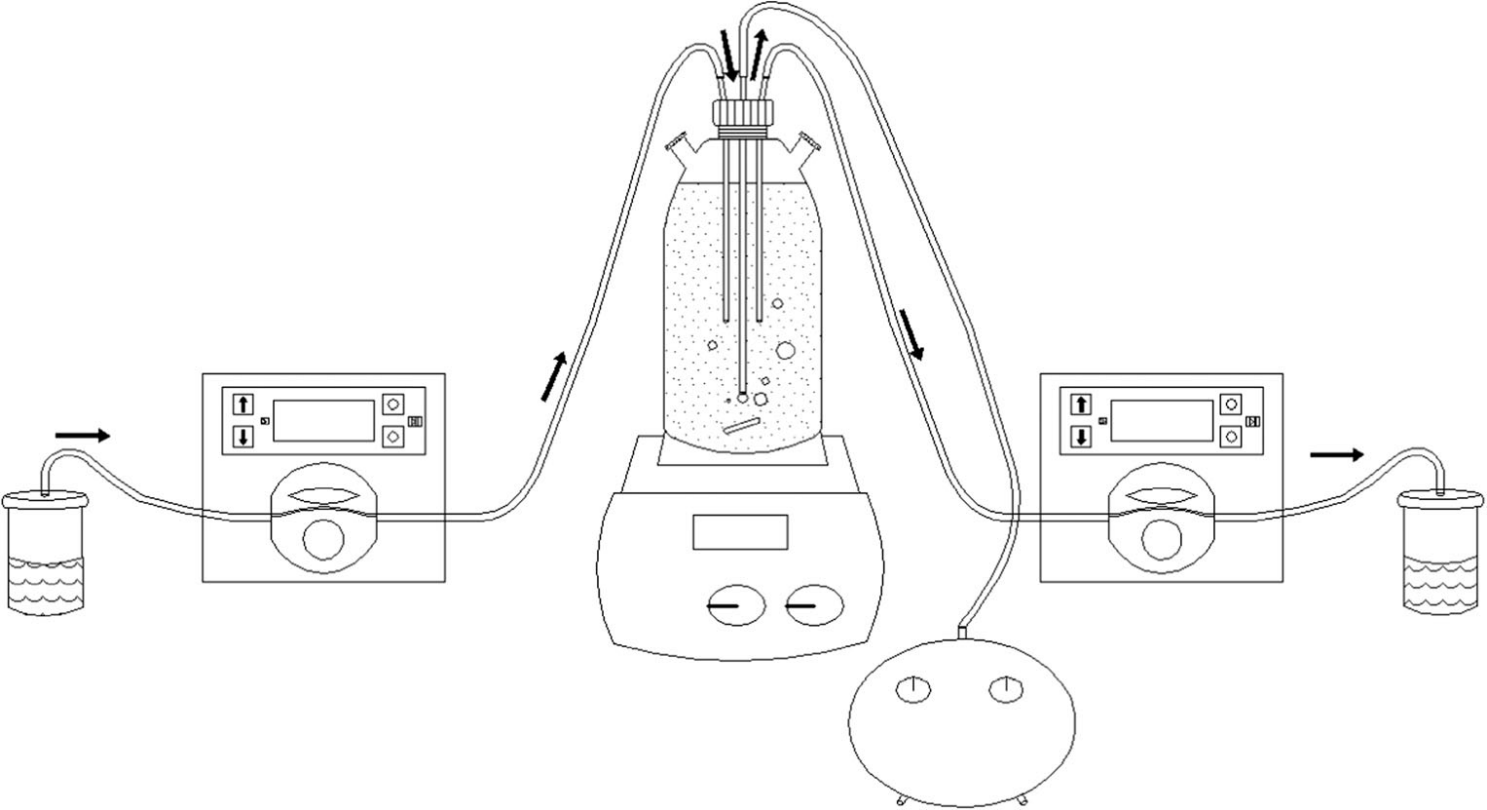
Research station

The culture was conducted in 1.0 dm^3^ (active volume) glass photobioreactors (SIMAX) placed on magnetic agitators with a set of lamps (NARVA, 2·36 W). The culture temperature was maintained at 25 ± 2 °C. Each reactor was equipped with an aeration system consisting of membrane pumps and diffusers distributing the supplied air (Fig. 2). The photobioreactors were illuminated at 3000 lx and aeration intensity was 0.6 vvm. Initial concentrations of microalgae biomass, characterised by the content of dry matter in the bioreactors, were prepared at the level of about 50 mg TS/dm^3^. Effluent dosing and the receipt of inoculum was carried out using peristaltic pumps (MasterFlex, 7525-20).

### Post-fermentation effluent

The experiment tested the effluent obtained from a UASB-type model anaerobic bioreactor, supplied with wastewater prepared based on acidic whey. The anaerobic bioreactor of a labyrinth flow and active volume of 70 dm^3^ worked under mesophilic conditions, at the load maintained at *A* = 2.6 kg BOD_5_/m^3^, hydraulic stop time of 15 days and the process temperature of 35 ± 2 °C. The values of the basic indices of the effluent and the synthetic culture medium are presented in Table 1. Post-fermentation effluent was subjected to vacuum microfiltration each time to remove the suspension which hindered light access to the culture. In the series control algae were cultured in Bold’s basal medium with threefold nitrogen and vitamins; modified (3N-BBM+V). The 3N-BBM+V compositions were as follows: NaNO_3_ 750 mg/dm^3^, CaCl_2_·2H_2_O 25 mg/dm^3^, MgSO_4_·7 H_2_O 75 mg/dm^3^, K_2_HPO_4_·3 H_2_O 11.5 mg/dm^3^, KH_2_PO_4_ 26.9 mg/dm^3^, NaCl 25 mg/dm^3^, Na_2_EDTA 0.0045 mg/dm^3^, FeCl_3_·6 H_2_O 0.582 mg/dm^3^, MnCl_2_·4 H_2_O 0.26 mg/dm^3^, ZnCl_2_ 0.03 mg/dm^3^, CoCl_2_·6 H_2_O 0.012 mg/dm^3^, NaMoO_4_·2 H_2_O 0.24 mg/dm^3^, B_12_ 1 ml/dm^3^ and B_1_ 1 ml/dm^3^.

**Table 1:** Characteristics of a post-fermentation effluent and synthetic medium

Index	Unit	Post-fermentation effluent	Synthetic medium
COD	mg O_2_/dm^3^	825	–
TN	mg/dm^3^	168.2 ± 5.5	109.6 ± 0.89
PO_4_^3−^ − *P*_total_	mg/dm^3^	10.08 ± 0.15	8.29 ± 0.15
Mn	mg/dm^3^	0.931 ± 0.02	< 0.02
Zn	mg/dm^3^	0.134 ± 0.01	< 0.005
Fe	mg/dm^3^	1.06 ± 0.02	< 0.1
SO_4_^2−^	mg/dm^3^	55.45 ± 2.1	7.31 ± 0.63
Reaction	–	8.5	7.79

### Experiment

The experimental research was divided into two phases: an adaptation phase and a flow culture. The cultivation was performed in three repetitions. At the same time, a control was carried out using a synthetic culture medium.

#### Adaptation phase

The adaptation phase was carried out in a photobioreactor filled with a culture medium and an addition of inoculum (50 mg/dm^3^). This adaptation culture was conducted in a stationary mode. Undiluted post-fermentation effluent was used as the culture medium. The aim of this phase was the adaptation of microalgae to the applied culture medium and biomass multiplication. This phase lasted until the maximum value of the biomass growth index was obtained. The culture was then continued in a flow culture mode (second phase). During the adaptation, the maximum daily total nitrogen removal was determined, which was the basis for determination of the daily dose of post-fermentation effluent supplied to the system. The maximum daily consumption of nitrogen compounds was from 16.55 ± 0.15 mg N/dm^3^/day. In the control series, the maximum daily consumption of nitrogen compounds was from 15.91 ± 0.99 mg N/dm^3^/day.

#### Flow culture

The second experimental phase involved the operation of the culture with dosing portions of post-fermentation effluent and the removal of the culture medium. The amount of nitrogen compounds introduced to the system with the culture medium was adjusted to be approximate to the daily nitrogen consumption in the adaptation culture (16.55 ± 0.15 mg N/dm^3^/day). Considering the content of the total nitrogen in the post-fermentation effluent, which was 168.2 ± 5.5 mg N/dm^3^, the daily dose was set at 96 cm^3^/day (2 cm^3^/0.5 h), which corresponded to the nitrogen amount at the level of 16.15 ± 0.53 mg N/dm^3^/day. In the control series, the flow was set at 145 cm^3^/day (3 cm^3^/0.5 h).

### Measurement of biomass concentration

The dry matter content (TS) was determined by filtering 20-ml samples of the culture through a 90 mm in diameter hard cellulose filter. Following the filtration process, the filter was dried in a laboratory dryer (Binder, Germany) until a stable mass was obtained. In order to determine the content of dry matter, we get the difference in mass between a dry filter before filtration and a dry filter after filtration. Measurements of dry matter content in the adaptation phase were done every 24 h and every 48 h during the flow culture phase.

### Determination of growth parameters and nitrogen removal parameter

The biomass productivity (*P*_biomass_, g TS/dm^3^/day) and nitrogen removal efficiency (*P*_nitrogen,_ mg N/dm^3^/day) were calculated based on the following equation:

$$ {P}_{\mathrm{biomass}/\mathrm{nitrogen}}=\frac{\varDelta X}{\varDelta t} $$

where Δ*X* is the difference in biomass concentration (g TS/dm^3^) or nitrogen concentration (mg N/dm^3^) over a cultivation time of Δ*t* (*d*).

### Taxonomic analysis

A taxonomic analysis of algae biomass was carried out under microscopic magnifications: ×1.25 ×10 ×40 or ×1.25 ×10 ×10 of an MF 346 (OPTA-TECH) biological microscope with 3-MP camera (Opta-Tech).

### Measurement of nutrient concentrations

To determine the biogenic compound removal efficiency in the flow culture of algae, a daily analysis was made of the total nitrogen and phosphorus at the discharge from the photobioreactors. The samples were pre-filtered through cellulose filters to remove solids. The filtered samples were subject to analyses using LCK cuvette tests (Hach Lange, USA).

### Statistical analysis

Statistical analysis was performed using Statistica software. The results are presented in the form of mean values ± standard deviation from using one-way analysis of variance (ANOVA). A difference was considered statistically significant at *p* < 0.05.

## Results and discussion

### Cell growth

Scientific references provide many examples of algae-based systems in wastewater treatment. Sawayama et al. (1995) used algae cultures of *Botryococcus braunii* species as the third phase of wastewater treatment in closed systems. Such a technological solution allowed for efficient removal of both nitrogen and phosphorus from communal wastewater discharged from activated sludge reservoirs. The use of wastewater resulted in algae biomass production with a high concentration of carbohydrates. Chiu et al. (2015) presented data and a detailed description of the application of varied waste-water in *Chlorella* sp. algae culture. They distinguish three main wastewater sources: communal, agricultural and industrial, containing a wide variety of components. Researchers indicate that nutrients such as nitrogen and phosphorus contained in wastewater may successfully be used as a culture medium for an intensive biomass culture.

In the present experiment, the *C. vulgaris* microalgae culture was conducted with the use of post-fermentation effluent as a culture medium. An undiluted post-methane fermentation effluent from the dairy industry was used. The experiment was divided into two phases. In the adaptation phase, the biomass was cultured in a culture medium in stationary mode until the maximum biomass productivity was obtained. In the flow phase, portions of culture medium were fed and portions of the effluent were removed. The initial concentration of biomass in the adaptation phase was 50 ± 12 mg TS/dm^3^. The maximum biomass productivity was obtained on day 12 of the culture and it was 413.67 ± 4.51 mg TS/dm^3^/day. On day 13, the productivity remained at a similar level (409.33 ± 3.51 mg TS/dm^3^/day), while on day 14, it dropped to 372 ± 4.51 mg TS/dm^3^/day. At that time, the experiment passed to the second phase (flow culture). The maximum biomass concentration, which was obtained in the adaptation phase, was 2.915 ± 17 mg TS/dm^3^. In the flow phase, the biomass concentration at the discharge from a photobioreactor remained at a similar level as in the adaptation phase, which confirms that the retention time of the effluent in the photobioreactor was well adjusted. The biomass content in the flow system ranged from 2.863 to 3.065 mg TS/dm^3^ at an average concentration of 2.953 ± 49 mg TS/dm^3^.

**Fig. 3:**
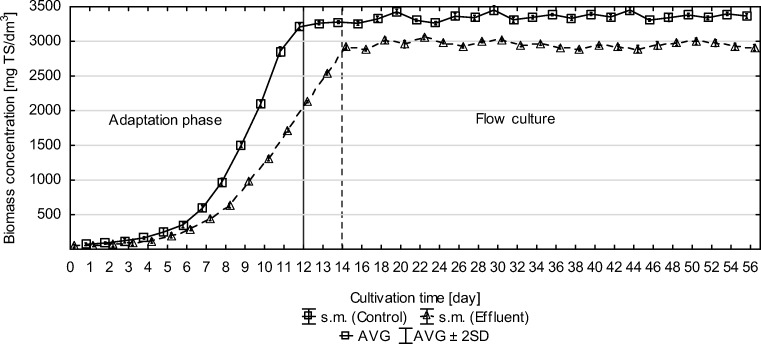
Biomass concentration in the adaptation phase and flow phase

In the control series, the initial concentration of biomass in the adaptation phase was 56.3 ± 11 mg TS/dm^3^. The maximum biomass productivity was obtained on day 11 of the culture and it was 757.67 ± 15.3 mg TS/dm^3^/day. The maximum biomass concentration, which was obtained in the adaptation phase was 3285 ± 9 mg TS/dm^3^. In the control series, the biomass content in the flow system ranged from 3254 to 3474 mg TS/dm^3^ at an average concentration of 3355 ± 52 mg TS/dm^3^ (Fig. 3).

Zhou et al. (2018) studied the possibility of culturing *Chlorella zofingiensis* using communal wastewater and post-methane fermentation effluent (swine slurry), mixed in varied proportions, as a culture medium. For the culture medium consisting of only communal wastewater, the biomass productivity was 280 mg/dm^3^/day. The maximum biomass productivity was obtained by adding 8% of anaerobic effluent to communal wastewater, which was used as a culture medium. This productivity was 630 mg/dm^3^/day. Zhu et al. (2013) carried out *C. zofingiensis* microalgae culture using diluted wastewater from swine production. The maximum biomass productivity achieved by them was 296.16 ± 19.16 mg/dm^3^/day. On the other hand, Sepúlveda et al. (2015) used effluent from anaerobic treatment of municipal waste at different dilution degrees (0–80%). The maximum biomass growth in this study reached 400 mg/dm^3^/day.

### Nutrient concentrations

The initial content of the total nitrogen in the culture medium in the adaptation phase was 168.2 ± 5.5 mg N/dm^3^. The highest daily consumption of nitrogen compounds in the adaptation phase was 16.55 ± 0.15 mg N/dm^3^/day. The dose of the supplied effluent was determined based on the daily consumption of nitrogen compounds, at the maximum biomass productivity, and was 96 cm^3^/day (2 cm^3^/0.5 h), which corresponded to the load of 16.15 ± 0.53 mg N/dm^3^/day. The total nitrogen concentration at the transition to the flow culture phase of the experiment was 10.68 ± 0.75 mg N/dm^3^. The total nitrogen concentration at the discharge from the photobioreactor during the flow phase ranged from 5.32 to 13.14 mg N/dm^3^ at an average concentration of 9.85 ± 1.49 mg N/dm^3^. The average efficiency of nitrogen compound removal in the flow culture was 15.66 ± 1.15 mg N/dm^3^/day.

**Fig. 4:**
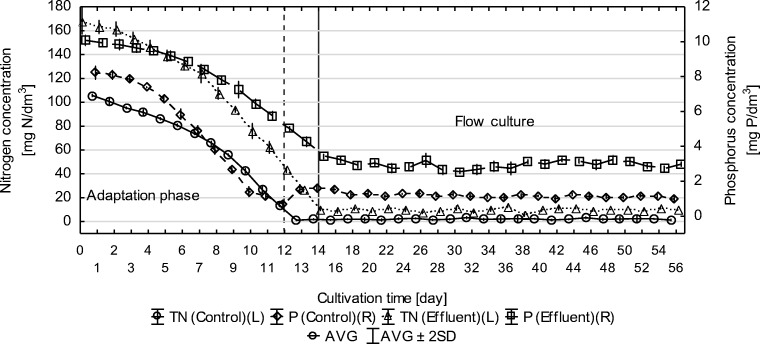
Total nitrogen and total phosphorus concentration in the adaptation phase and flow phase

In the control series, the initial content of the total nitrogen in the culture medium in the adaptation phase was 109.60 ± 0.89 mg N/dm^3^. The highest daily consumption of nitrogen compounds in the adaptation phase was 15.91 ± 0.99 mg N/dm^3^/day. In the control, the total nitrogen concentration at the discharge from the photobioreactor during the flow phase ranged from 0.92 to 3.75 mg N/dm^3^ at an average concentration of 2.05 ± 0.73 mg N/dm^3^. The average efficiency of nitrogen compound removal in the flow culture was 15.78 ± 0.50 mg N/dm^3^/day (Fig. 4).

The initial content of the total phosphorus in the culture medium in the adaptation phase was 10.08 ± 0.15 mg P/dm^3^. The total phosphorus concentration in the transition to the flow culture was 3.42 ± 0.08 mg P/dm^3^. The average efficiency of phosphorus compound removal in the flow phase was 0.84 ± 0.12 mg P/dm^3^/day.

In the control series, the initial content of the total phosphorus in the culture medium in the adaptation phase was 8.28 ± 0.15 mg P/dm^3^. The average efficiency of phosphorus compound removal in the flow phase was 1.15 ± 0.08 mg P/dm^3^/day (Fig. 4).

Zhou et al. (2018) obtained the highest degree of total nitrogen removal using only communal wastewater as the culture medium (21 mg N/dm^3^/day). The removal degree of phosphorus was 4.6 mg P/dm^3^/day. Sepúlveda et al. (2015), in their experiment involving post-methane fermentation effluent from communal wastewater obtained the maximum degree of nitrogen removal of 35 mg N/dm^3^/day. The efficiency of phosphorus removal was 5.7 mg P/dm^3^/day. Cabanelas et al. (2013) decided to use effluents originating from varied phases of treatment of the municipal wastewater treatment station to culture *C. vulgaris*. As the result of the experiment, they obtained the maximum nitrogen and phosphorus removal index of 9.8 mg N/dm^3^/day and 3.0 mg P/dm^3^/day, respectively. However, Sevrin-Reyssac (1998) used swine slurry to grow a polyculture consisting of *Scenedesmus falcatus*, *Scenedesmus quadricauda*, and *Chlorella* sp*.* and the maximum degree of nitrogen removal was 12.0 mg N/dm^3^/day.

Effluent from anaerobic fermentation of acid whey was used in the study. The study proved that cultivation of *C. vulgaris* with a capacity of 1 m^3^ annually will allow managing of 35 m^3^ of acid whey.

## Conclusions

High efficiency in biogenic compound removal had a positive effect on the final biomass content of the tested microalgae. The application of the tested wastewater considerably reduced the necessity of using chemical reagents. The content of nitrogen and phosphorus in wastewater was sufficient for conducting an effective culture of algae. The efficiency of nitrogen removal in the flow system was 15.61 ± 1.38 mg N/dm^3^/day.
